# Evictions and Infant and Child Health Outcomes: A Systematic Review

**DOI:** 10.1001/jamanetworkopen.2023.7612

**Published:** 2023-04-11

**Authors:** Bruce Ramphal, Ryan Keen, Sakurako S. Okuzuno, Dennis Ojogho, Natalie Slopen

**Affiliations:** 1 Harvard Medical School, Boston, Massachusetts; 2 Harvard T.H. Chan School of Public Health, Boston, Massachusetts; 3 Harvard Law School, Cambridge Massachusetts; 4 Center on the Developing Child, Cambridge Massachusetts

## Abstract

**Importance:**

Millions of rental evictions occur in the United States each year, disproportionately affecting households with children. Increasing attention has been paid to the impact of evictions on child health outcomes.

**Objective:**

To synthesize and assess studies examining the associations of eviction exposure with infant and child health outcomes.

**Evidence Review:**

For this systematic review without meta-analysis, a database search was performed using PubMed, Web of Science, and PsycINFO, through September 25, 2022. Included studies were peer-reviewed quantitative studies examining an association between exposure to eviction and at least 1 health outcome, both before age 18 years, including prenatal exposures and perinatal outcomes. This study followed the Preferred Reporting Items for Systematic Reviews and Meta-analyses (PRISMA) reporting guideline. Data were analyzed from March 3 to December 7, 2022.

**Findings:**

Database searches identified 266 studies, and 11 studies met inclusion criteria. Six studies examined associations between prenatal eviction and birth outcomes, such as gestational age, and each found that eviction was significantly associated with at least 1 adverse birth outcome. Five studies investigated other childhood outcomes, including neuropsychological test scores, parent-rated child health, lead testing rates, and body mass index, and among these 5 studies, 4 reported an association between eviction and adverse child health outcomes. Direct experience of eviction or residence in a neighborhood with more evictions was associated with adverse perinatal outcomes in 6 studies, higher neurodevelopmental risk in 2 studies, worse parent-rated child health in 2 studies, and less lead testing in 1 study. Study designs and methods were largely robust.

**Conclusions and Relevance:**

In this systematic review without meta-analysis of the association between evictions and child health outcomes, evidence demonstrated the deleterious associations of eviction with a range of developmental periods and domains. In the context of a rental housing affordability crisis, ongoing racial disparities in evictions, and continuing harm to millions of families, health care practitioners and policy makers have an integral role to play in supporting safe, stable housing for all.

## Key Points

**Question:**

Are residential evictions or community-level eviction rates associated with infant and child health outcomes?

**Findings:**

This systematic review surveyed 11 studies reporting associations between direct experience of and proximity to evictions and adverse birth outcomes, such as preterm birth and low birthweight. Evidence suggests that childhood exposure to evictions was associated with harms to neurodevelopment and overall child health.

**Meaning:**

These findings suggest that eviction prevention may contribute to improved health outcomes in affected communities.

## Teaser

This systematic review without meta-analysis synthesizes and assesses data from studies examining the associations of eviction exposure with infant and child health outcomes.

## Introduction

Housing conditions affect children’s health profoundly, and deterioration in the stability or quality of housing worsens child health.^1^ For example, children who have experienced multiple moves or homelessness are more likely to develop various health conditions, including mental illnesses,^2^ respiratory conditions,^3^ and infections.^3^ One phenomenon that contributes to housing insecurity and may be uniquely harmful to children and their communities is eviction. Each year in the United States, more than 2 million eviction case filings occur, yielding nearly 1 million completed evictions.^4,5^ As many as 5.5 times more informal evictions occur annually, in which landlords evict tenants extralegally.^6^ Generally, eviction procedures involve a written notice from a landlord communicating their intention to end a tenant’s lease, followed by a court filing, trial, and judgment. Whereas most landlords have attorneys, few tenants do, although some municipalities have made free legal counsel available to low-income households facing eviction.^7,8^ Landlords may initiate eviction proceedings to remove tenants who have not paid rent or who have violated their leases but can also do so for reasons entirely out of a tenant’s control (eg, sale of property). Households with children are more likely to experience evictions.^9^ In the context of racial and ethnic income and wealth inequality, Black and Latinx women and children are especially at risk for eviction.^10,11^ Notably, these groups are more likely to be evicted even when controlling for income, suggesting inadequate enforcement of antidiscrimination laws.^12,13,14,15^ Disparities in the threat of eviction have persisted during the COVID-19 pandemic, which has disproportionately impacted the health and financial security of American Indian, Black, and Latinx families.^16,17,18^

Evictions disrupt nearly every facet of a family’s life and are associated with exerting multiple negative influences on child health and development via several putative pathways. Prenatal stress is associated with harms in maternal and fetal physiology and adverse birth outcomes, such as preterm birth.^19^ Both prenatal and early life stress have been associated with increased risk of pediatric mental health problems^20^ and reduced executive functioning.^21^ Furthermore, eviction creates acute financial precarity,^22^ potentially compromising parental well-being and access to nutritious food, medication, and health care; all of these sequelae are associated with increased risk of poor child outcomes.^23^ In addition, as suggested by the Family Stress Model,^24^ this economic hardship and associated stress can negatively impact parental relationships with each other and their children.^25^ Involvement in formal eviction proceedings, even when groundless, typically remains on a tenant’s rental history for years, making them less likely to be approved for future rental applications. Evicted families are likely to move to lower-quality housing, increasing exposure to allergens, air pollution, toxicants, and unsafe homes.^26,27^ Therefore, an eviction may affect child health both through its acute effects and its cascading consequences that span multiple stages and settings of child development.

Evictions are not only harmful to families and children, but also to the communities in which they occur.^28,29,30,31,32,33,34^ Communities with elevated rates of eviction are more likely to endure adversities that harm the health of residents, including those who do not directly experience displacement. For example, spatially clustered evictions are associated with higher neighborhood violence^28,29,30,31^ and increased spread of communicable disease^29^ and may also contribute to stress by portending housing insecurity for neighboring renters and disrupting neighborhood social dynamics.^32^

A 2017 systematic review by Vásquez-Vera et al^35^ surveyed the health outcomes associated with evictions among individuals in rented and owned homes. At the time of their review, the authors found only 1 quantitative study that included children.^23^ Since then, the link between evictions and child health has received increased research attention. Here, we systematically review quantitative studies examining associations between exposure to rental evictions and child health outcomes. We expected the literature on evictions and child health to support the hypothesis that evictions, which deprive families of a basic need, were associated with harm to child health across a wide range of organ systems and developmental periods. We intend to summarize existing knowledge, evaluate the quality of evidence, and situate this research within the ongoing housing affordability crisis,^36^ in which 5.7 million children are in households that are behind on rental payments as of July 2022.^37^ Moreover, rental evictions disproportionately affect structurally marginalized populations, such as American Indian, Black, and Latinx families,^16^ and we discuss the role that policy, physician advocacy, and patient-tenant empowerment can play in supporting housing, reproductive, and health justice.^17^

## Methods

### Search Strategy and Eligibility Criteria

For this systematic review without meta-analysis, we conducted an electronic literature search using PubMed, Web of Science, and PsycINFO to identify relevant studies published from database inception through September 25, 2022. Search strategies are provided in eAppendix 1 in Supplement 1. We also manually searched the reference lists of identified articles. We included peer-reviewed quantitative studies using the following inclusion criteria: (1) at least 1 health outcome, (2) age younger than 18 years, (3) quantitative analyses, and (4) exposure variable limited to direct eviction or neighborhood-level eviction rates. Exposure to rental eviction was assessed either as a direct experience of eviction or based on neighborhood-level eviction rates. We defined child health outcomes broadly to include physical and mental health, cognitive outcomes, birth outcomes, health behaviors, health care utilization, and health care access. We registered our search on PROSPERO prior to abstract review (CRD42022301849). This review follows the Preferred Reporting Items for Systematic Reviews and Meta-analyses (PRISMA) reporting guideline.

### Study Selection, Data Extraction, and Synthesis

Two independent reviewers (B.R. and N.S.) assessed studies identified by our searches based on titles and abstracts. We reviewed potentially relevant articles in detail for adherence to inclusion criteria, and disagreements were discussed and resolved. Three investigators (B.R., R.K., and S.S.O.) independently extracted data from the studies (2 investigators per study), including study design, location, sample size, participant demographics, recruitment strategy, eviction measures, health outcomes, statistical methods, and results. We used a qualitative approach to synthesizing included studies; a meta-analysis was not possible due to the small number of studies and variability in outcomes. For presentation of the results, we organized the included studies into 2 groups: birth outcomes and later health outcomes.

### Quality Assessment

The quality of evidence of each study was quantified using an adapted scoring system based on the Newcastle-Ottawa scale^38^ (eAppendix 2 in Supplement 1). Three investigators (B.R., R.K., and S.S.O.) independently rated studies based on the following criteria: (1) representativeness of the cohort experiencing eviction (1 point); (2) recruitment of a nonexposed cohort that is comparable in location or age and sex (1 point); (3) absence of health outcome at start of study (1 point); (4) adequacy of methods to reduce confounding (2 points); (5) measurement of the outcome by researchers, records, or validated instrument (1 point); (6) clarity of statistical reporting (1 point); and (7) adequacy of methods to address bias due to missing data (1 point). We awarded 2 points for approaches to reduce confounding if the study controlled household socioeconomic status and used additional robust approaches to reduce confounding, such as propensity scores, fixed effects, or marginal structural models. Therefore, scores could range from 0 to 8, with higher score indicating higher quality of evidence. Disagreements in scoring were discussed and resolved via consensus. Data were analyzed from March 3 to December 7, 2022.

## Results

### Search Results

Our database search yielded 289 studies (Figure). After title and abstract screening, 27 full-text articles were assessed for inclusion; of these, 11 studies^23,32,33,34,39,40,41,42,43,44,45^ met all inclusion criteria. The reference list search yielded no additional studies.

**Figure.**
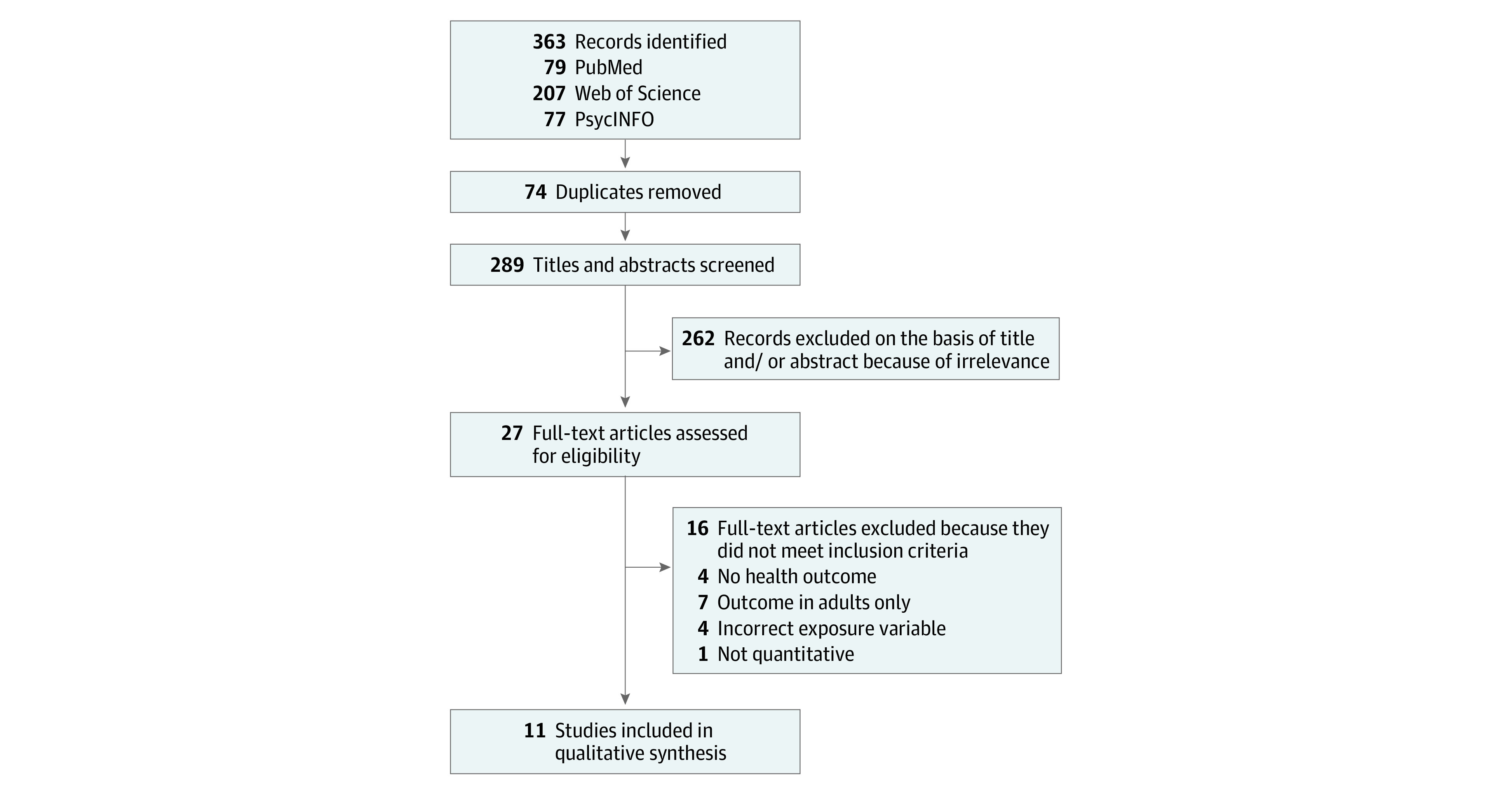
Flowchart of Study Selection for the Identification of Peer-reviewed Articles on Housing Eviction and Child Health in the United States

Ten of the 11 included studies^32,33,34,39,40,41,42,43,44,45^ were published after 2020 (Table 1). Five studies^23,32,41,42,44^ examined multistate or national samples, 1 study^39^ was statewide, and 4 studies^33,34,40,43^ were regional. Five studies^32,33,34,39,45^ constructed retrospective cohorts (ie, secondary analysis of cohort data), 3 studies^23,41,42^ used a longitudinal design, 2 studies^43,44^ were cross-sectional, and 1 study^40^ used an ecological design. Sample sizes ranged from 1386 to 7 324 812 participants; the median was 19 096 participants. Six studies^32,33,34,39,40,45^ examined birth outcomes, and the remaining 5 studies examined cognitive or neurodevelopmental outcomes,^42,44^ parent-reported general child health,^23,44^ weight,^41,44^ and lead testing.^43^ Six studies^23,39,41,42,43,44^ defined eviction exposure based on participant experiences of eviction, and 5 studies^32,33,34,40,45^ used neighborhood-level eviction rates. One study examined informal evictions.^44^ Table 2 contains study details.

**Table 1.** Peer-reviewed Studies of Housing Eviction and Child Health

Study characteristics	No. of studies (%) (N = 11)
Year of publication	
Before 2020	1 (9)
2020-2021	10 (91)
Geographic region	
Multistate or national	6 (55)
Statewide	1 (9)
Regional	4 (36)
Study design	
Retrospective^a^	5 (45)
Longitudinal	3 (27)
Cross-sectional	2 (18)
Ecological cross-sectional	1 (9)
Sample sizes^b^	
1386-19 096	5 (45)
>19 096	5 (45)
Not applicable^c^	1 (9)
Child ages at outcome	
Birth	6 (55)
Early childhood (0-5 y)	3 (27)
Middle childhood (6-11 y)	1 (9)
Adolescence (12-18 y)	1 (9)
Eviction data	
Individual level	6 (55)
Neighborhood level	5 (45)
Health outcomes examined	
Birth outcome	6 (55)
Fair or poor parent-rated child health	2 (18)
Anthropometric	2 (18)
Blood lead testing	1 (9)
Blood lead levels	1 (9)
Cognitive/neurodevelopmental outcomes	2 (18)

^a^ Retrospective design refers to studies that analyzed historical data on eviction history.

^b^ The median sample size was 19 096 participants.

^c^ One study had an ecological design.

**Table 2.** Characteristics of Studies Used to Assess Housing Eviction in Relation to Children’s Health Outcomes

Source	Design	Population (No. of participants)^a^	Measure of housing eviction (report or administrative)	Health outcomes (mode of assessment)	Approaches to address confounding and any tests for association modification or mediation	Major findings	Evidence grade
Birth outcomes							
Khadka et al,^32^ 2020	Retrospective cohort	All singleton live births from 2009-2016 in 1633 US counties (7 324 812)	County eviction filing rate during pregnancy	Preterm birth, gestational age, low birthweight status (administrative data)	Control for observed covariates, placebo test,^b^ stratified by race and Medicaid status	Higher eviction rates during pregnancy, particularly during the second and third trimester, were associated with a 1.09–percentage point increase in the risk of preterm birth and a 0.72–percentage point increase in the risk of low birthweight. Heterogeneity in associations were observed based on race and ethnicity and Medicaid payment status, and estimates were imprecise across racial and ethnic subgroups and Medicaid payment status categories.	8
Hazekamp et al,^40^ 2020	Ecological	Census tract birth data from 2013-2017 in Chicago, Illinois	Census tract eviction rate (filings and judgments) from 2012-2016	Low birthweight rate, infant mortality rate (administrative data)	Control for observed covariates	Census tracts with higher eviction filing rates had higher low birthweight rates (β = 0.12 [95% CI, 0.08 to 0.17]) and infant mortality rates (β = 0.360 [95% CI, 0.07 to 0.65]); similar, but more pronounced patterns were observed for estimated associations with tract-level eviction rate.	6
Sealy-Jefferson et al,^33^ 2021	Retrospective cohort	Birth cohort of African American women in Detroit, Michigan (1386)	Census block group eviction rate (filings and judgments)	Preterm birth (hospital record)	Control for observed covariates, marital or cohabiting status examined as moderator	Among married or cohabiting women only, preterm birth risk was associated with tract-level eviction filing rate (RR, 1.25 [95% CI, 1.06 to 1.47]) and eviction judgement rate (RR, 1.18 [95% CI, 1.05 to 1.33]).	6
Freedman et al,^34^ 2022	Retrospective cohort	Hospital EHR-based cohort of singleton births from 2008-2018 in Chicago, Illinois (119 010)	Census block group eviction rate (filings and judgments)	Preterm birth, small for gestational age, placental lesions (hospital record)	Control for observed covariates, fixed effects, stratified by neighborhood median household income, insurance type, and birth before or after housing crisis (2008-2012)	Compared with pregnant individuals residing in block groups in the lowest quartile, those in block groups in the highest quartile for eviction filing rate had greater risk of delivering preterm (OR, 1.17 [95% CI, 1.08 to 1.27]) and delivering a small-for-gestational-age infant (OR, 1.13 [95% CI, 1.03 to 1.25]). Associations were specific to lower-income neighborhoods, more pronounced among Black families, and during the housing crisis. No associations observed for placental lesions.	7
Himmelstein et al,^39^ 2021	Retrospective cohort	All children born in Georgia from 2000-2016 whose mother experienced an eviction filing pre- or postnatally (88 862)	Linked eviction filing records during or after pregnancy	Birthweight, low birthweight status, infant mortality, gestational age, preterm birth (administrative data)	Control for observed covariates, fixed-effects, stratified analyses by race, marital status, urban vs rural residence, and maternal education	Eviction filing during pregnancy associated with lower birthweight (difference, −26.88 [95% CI, −39.53 to 14.24] g), higher rates of low birthweight (difference, 0.88% [95% CI, 0.23% to 1.54%]), lower gestational age (difference, −0.09 [95% CI, −0.16 to −0.03] wk), and higher rates of preterm birth (difference, 1.14% [95% CI, 0.21 to 2.06]), relative to neonates of mothers who experienced an eviction action at a time outside of the pregnancy. Associations were most pronounced among unmarried mothers and similar among Black and White participants.	8
Harville et al,^45^ 2022	Retrospective cohort	All live births in 2016 across 5924 counties in 45 states (2 950 965)	County eviction filing rate in 2015	Low birthweight, preterm birth (administrative data)	Control for observed covariates, stratified analysis by maternal race and ethnicity	Infants born to Black and White women in the counties in the highest quartile of eviction rate had an increased risk of low birthweight (Black women: OR, 1.17 [95% CI, 1.10 to 1.25]; White women: OR, 1.05 [95% CI, 1.02 to 1.09]) and preterm birth (Black women: OR, 1.14 [95% CI, 1.06 to 1.24]; White women: OR, 1.07 [95% CI, 1.03 to 1.11]); similar pattern of results observed in analyses using quartiles of eviction filing rate. Associations not evident among infants born to Hispanic women.	7
Childhood health outcomes							
Leifheit et al,^41^ 2020	Longitudinal	FFCWS birth cohort (1928, 2970 at age 9 y, and 940 at age 15 y)	Mother-reported eviction in 12 mo before child age 1, 3, or 5 y	Obesity status, measured as BMI greater than within-sample 95th percentile, measured during follow-up visit at age 15 y	Control for observed covariates, inverse probability of treatment weighting	No association between eviction in early childhood and later obesity status.	7
Schwartz et al,^42^ 2021	Longitudinal	FFCWS birth cohort with eviction data (1724 in infancy, 2126 in early childhood, and 1979 in middle childhood)	Mother-reported eviction in 12 mo before infancy (1 or 2 y), early childhood (3 or 5 y), or middle childhood (9 y) follow-up visits	Immediate and working memory (WISC-IV Digits Forward & Backward Standard Score), math word problems (WJ-III Applied Problems), reading comprehension (WJ-III Passage Comprehension), vocabulary (Peabody Picture Vocabulary Test) at age 9 y	Control for observed covariates, inverse probability of treatment weighting	Eviction during middle childhood associated with worse working memory (β = −0.25 [95% CI, −0.48 to −0.02]), worse performance on math word problems (β = −0.43 [95% CI, −0.76 to −0.10]), and more limited vocabulary (β = −0.39 [95% CI, −0.65 to −0.12]) at age 9 y.	7
Desmond et al,^23^ 2015	Longitudinal	Children from the 20-city FFCWS birth cohort (2676)	Mother-reported eviction in 12 mo before child age 3 or 5 y	Mother-reported child health status (single-item, dichotomized to fair or poor vs good, very good or excellent) at age 5 y	Control for observed variables, propensity score matching, placebo test,^b^ fixed effects	Recent eviction was associated with worse mother-reported child health status (ie, in a model with propensity score weighting to examine recent eviction, the weighted logit coefficient estimate is equivalent to a 0.10 [SE, 0.05] difference (in the probability of child’s poor health).	6
Richter et al,^43^ 2021	Cross-sectional	Households with children who were threatened with eviction in Cleveland, Ohio, from 2013-2015 (11 750)	Whether an eviction filing resulted in move-out date, a proxy for eviction judgment	Lead testing by age 2 y, elevated blood lead status	None	Lead testing frequency was lower for children with an eviction filing (52.6%), or an eviction move-out order (48.5%) relative to the estimate for the city overall (65.9%); among those tested, elevated levels were nearly twice as common among those with an eviction filing (>5 μg/DL: 17.1%; >10 μg/DL: 5.6%) or with an eviction move-out (>5 μg/DL: 17.7%; >10 μg/DL: 5.9%) compared with the estimate for the city overall (>5 μg/DL: 9.9%; >10 μg/DL: 3.0%).	2
Cutts et al,^44^ 2022	Cross-sectional	ED- and primary care–based cohort in 4 US cities from 2011-2019 (26 441)	Mother-reported eviction in past 5 y, specified whether formal or informal	Mother-reported child health status (single item, dichotomized to fair or poor vs good or excellent); hospital admission from ED visit, developmental risk, underweight risk, obesity risk, ever hospitalized, all measured at median child age 19.9 mo	Control for observed variables	Compared with children who did not experience formal or informal evictions, evictions were associated with elevated odds of development risk (OR, 1.55 [95% CI, 1.32 to 1.82]), fair or poor parent-rated child health (OR, 1.43 [95% CI, 1.17 to 1.73]), and hospitalization after an ED visit (OR, 1.24 [95% CI, 1.01 to 1.53]); estimates were less precise for anthropomorphic assessments.	5

Abbreviations: BMI, body mass index (calculated as weight in kilograms divided by height in meters squared); EHR, electronic health record; FFCWS, Fragile Families and Child Well-being Study; OR, odds ratio; RR, relative risk; WISC-IV, Weschler Intelligence Scale for Children Fourth Edition; WJ-III, Woodcock Johnson III Tests of Achievement.

^a^ For longitudinal studies, age at baseline is provided.

^b^ The term *placebo test* was used to refer to an analysis to test whether eviction was associated with child health prior to eviction.

### Eviction and Perinatal Outcomes

Six studies^32,33,34,39,40,45^ examined associations between eviction and birth outcomes, including preterm birth, gestational age, low birthweight, small for gestational age, infant mortality, and placental lesions. Across all studies, eviction was significantly associated with at least 1 adverse birth outcome. One study^39^ examined individual experiences of eviction in relation to birth outcomes; using a large administrative data set from Georgia, Himmelstein and Desmond found that experiencing an eviction filing during pregnancy was associated with an increased risk of preterm birth, low birthweight, shorter gestation. Five studies^32,33,34,40,45^ used neighborhood-based measures of eviction, ranging from census block–level to county-level eviction rates. Drawing on birth certificate data from 2009 to 2016, 1 study^32^ included 7.3 million singleton births from more than half of the counties in the United States and found that residing in a county with a higher eviction rate was associated with higher rates of preterm birth and low birthweight. This finding was corroborated in another national study examining 2.9 million births from 2016.^45^ Associations between neighborhood-based eviction filing rates and preterm birth,^34^ low birthweight,^40^ and infant mortality^40^ were also found in other studies examining smaller geographic areas.

Across birth outcome studies, there were some inconsistencies regarding household composition as an association modifier. For example, a study by Sealy-Jefferson et al^33^ included Black women recruited for a birth cohort at a Detroit, Michigan, hospital and found that the association between neighborhood eviction rates and preterm birth was only present among those who were married or cohabiting with the child’s father. In contrast, the study by Himmelstein and Desmond^39^ found that the association between experiencing an eviction and lower birthweight was only present among unmarried individuals. Studies also reported inconsistent evidence for association modification by race. A study by Freedman et al^34^ based in Chicago, Illinois, found that the association between eviction rate and preterm birth was more pronounced among Black participants compared with Hispanic and White participants, whereas a national study by Harville et al^45^ showed stronger associations among Black and White participants than among Hispanic participants. In contrast, the study in Georgia by Himmelstein and Desmond^39^ did not find differences in associations between eviction rate and birthweight among Black and White participants. A national study by Khadka et al^32^ produced mixed findings related to differences in associations based on race and ethnicity across various birth outcomes.

### Eviction and Child Health Outcomes

Five studies^23,41,42,43,44^ investigated associations between eviction and child health outcomes. Three studies^23,41,42^ used the Fragile Families and Child Well-being Study (FFCWS),^46^ a prospective birth cohort of nearly 5000 participants in 20 US cities. Notably, the FFCWS oversampled births to unmarried mothers. Therefore, the study population includes a high proportion of Black, Hispanic, and low-income families, which are demographic groups who disproportionately experience eviction.^10,11^ Participants regularly reported on their housing status, allowing longitudinal analyses of the associations of evictions with a variety of outcomes. One FFCWS study by Desmond and Kimbro^23^ found that a recent eviction was associated with worse mother-reported child health status at age 5 years based on a single Likert-style item within a questionnaire. Another FFCWS study by Leifheit et al^41^ observed no association between eviction before age 5 years and obesity at age 15 years, although families who experienced eviction had an increased risk of food insecurity. These findings related to mother-reported health status, obesity, and food insecurity were replicated in another study by Cutts et al^44^ of younger children (ie, age <4 years) with additional data on informal evictions. Importantly, Cutts et al^44^ also found that evicted children had more hospitalizations and worse access to energy, health care, and childcare.^44^

A third FFCWS analysis by Schwartz et al^42^ found that children aged 9 years who experienced an eviction in the year before testing had lower scores on assessments of working memory, math, and vocabulary.^42^ This aligns with the multicity study by Cutts et al^44^ that found that maternal history of eviction (formal or informal) in the past 5 years was associated with child developmental risk, a measure that quantifies child cognitive, motor, and behavioral milestones.^44^ Another study by Richter et al^43^ used an administrative data linkage to examine the frequency of lead testing and elevated blood lead levels among Cleveland, Ohio, households by eviction history. By age 2 years, the frequency of lead testing was lower and the frequency of lead poisoning was higher among children in households with an eviction filing relative to all children in Cleveland; when the eviction filing led to an eviction move-out order, lead testing was even less frequent.^43^

### Quality of Evidence

Across studies, the quality of evidence was strong. All but 2 studies^43,44^ received scores greater than or equal to 6 out of 8 possible points using an adapted version of the Newcastle-Ottawa rating scale.

## Discussion

This systematic review without meta-analysis identified 11 studies^23,32,33,34,39,40,41,42,43,44,45^ examining associations between evictions and infant and child health outcomes. The 6 studies^32,33,34,39,40,45^ focused on perinatal outcomes consistently found that direct experience of eviction or residing in a neighborhood with more evictions was associated with a higher risk of adverse perinatal outcomes, such as preterm birth or low birthweight. The 5 studies^23,41,42,43,44^ focused on child outcomes found that children with eviction exposure had lower scores on measures of infant developmental risk, academic performance, working memory, worse parent-rated child health, and possibly less lead testing, but not increased body mass index. The study designs and methods were largely robust, with many using longitudinal designs, advanced approaches to address potential confounding, and samples that were either representative of their local communities or enriched for communities at higher risk for eviction.

The reviewed studies advance the literature reporting an association between residential mobility and child outcomes,^47^ including birth outcomes,^48^ cognitive development,^49,50,51^ and general parent-rated child health.^49^ Notably, adverse birth outcomes are a risk factor associated with future eviction,^52^ suggesting a feedback loop linking eviction and poor health.^53^ The results of this review are complemented by longitudinal studies that have identified long-term associations of childhood eviction with adult health,^54,55^ illustrating that the health outcomes observed within the reviewed studies may extend to adulthood. A previous systematic review by Vásquez-Vera et al^35^ primarily comprised studies on adults and reported that evictions were associated with a range of physical and mental health conditions, including depression, anxiety, psychological distress, suicides, and high blood pressure.^35^ Although these outcomes have not been examined in children who have experienced eviction, to our knowledge, our review provides evidence that the deleterious associations of rental evictions with health begin in utero and extend throughout development. Given the role of prenatal and early childhood experiences in shaping adult risk for a wide range of conditions, including diabetes, cardiovascular disease, neurodegenerative disorders, cancer, substance use, depression, cognitive impairment, and immune derangement,^56^ eviction prevention could support not only child health, but also future adult health.

### Implications for Future Research

Future studies should consider how evictions are associated with a broader range of child health outcomes beyond the perinatal period and how these outcomes vary longitudinally. Developmentally informed research has the potential to inform prevention and intervention strategies. Many of the studies we reviewed leveraged the FFCWS, one of very few cohort studies that has prospectively collected high-quality data on evictions and focused on low-income mothers. Administrative data linkage approaches^39,53^ and publicly accessible neighborhood-level eviction metrics, such as the eviction-tracking map maintained by Eviction Lab,^57^ facilitate studying the outcomes associated with evictions among past, current, and future pediatric cohorts. Importantly, only 1 study^44^ collected data on informal evictions; future studies should examine informal evictions, which are much more common than formal evictions.^6^ Finally, in addition to quantitative studies, moving forward, complementary studies using qualitative methods may accelerate understanding of how sociological phenomena can serve as root causes of evictions and their heath sequelae. Considering the wide range of designs and populations, inconsistent findings regarding association modification may represent the effects of cohort composition, local tenants’ rights, regional variability in housing discrimination, or other sociopolitical dynamics.

### Implications for Practice and Policy

Because rental evictions are a common, powerful mechanism by which housing insecurity and homelessness occur, reducing evictions would likely have a meaningful impact on population health. As previously outlined,^58^ pediatricians, obstetrician-gynecologists, health systems, and local, state, and national policy makers all play essential roles in eviction prevention. Clinicians are uniquely poised to identify patients at risk of eviction and make referrals to public and community-based solutions. Screening and referral can improve outcomes associated with housing^59^ and child health,^60,61,62^ and various screening measures related to housing are validated for clinical practice.^63,64^ Given that many clinicians feel uncertain about how to address material hardship among patients,^65^ systematic protocols could empower clinicians to efficiently connect patients with local legal and housing resources.^62,63,65,66^

Health care systems can also bolster programs to support families with unexpected and chronic medical needs. For example, prospective research from the FFCWS found that infants in rental housing at the study’s baseline who had experienced an adverse birth outcome had an elevated risk of eviction through age 5 years.^52^ Innovative practices within health systems may involve creating partnerships with housing authorities and nonprofit organizations to directly connect families with safe, secure housing. For example, a 2020 pilot randomized trial found that integrated care models that include placement in affordable housing and financial, legal, and case management services lead to improvements in child health and parental mental health.^60^

At the policy level, enacting housing policies that offer resources to individuals at risk of eviction must be a priority.^58,67^ For example, several cities have recently expanded access to legal representation for low-income tenants facing eviction, which is proven to prevent displacement^7,68,69^; federal protections are needed to ensure that all tenants can access representation during eviction proceedings. Furthermore, housing voucher funding remains inadequate to meet the nation’s housing need.^70^ Whereas traditional methods of supporting housing security have modest associations with health outcomes,^61,71^ decisive, drastic, tenant-friendly housing policies have been associated with significant health outcomes in the COVID-19 pandemic. Specifically, eviction moratoria have been found to be associated with decreasing SARS-CoV-2 transmission,^29^ reducing COVID-19 mortality,^72^ and supporting adult mental health.^73^ Although this literature is nascent and has not examined child health, it is likely that proposed housing bills, such as the Eviction Crisis Act and the Family Stability and Opportunity Vouchers Act, would have beneficial outcomes in population health.^74^

In addition to directly protecting tenants from eviction, structural interventions that address the root causes of housing precarity would likely have health benefits. Fundamentally, the dearth of affordable housing facilitates evictions; thus, barriers to the construction of affordable multifamily housing, such as exclusionary zoning, should be dismantled.^75^ Other systemic issues, such as quality of education, income inequality, health care inaccessibility, insufficient worker protections and wages, unemployment, and inadequate paid sick and parental leave all contribute to the erosion of housing stability. For example, states that expanded Medicaid under the Affordable Care Act, an ostensibly nonhousing intervention, subsequently had fewer evictions^76^; thus, policies that address other forms of social inequality would likely support housing stability and health.

Specifically, structural racism, sexism, and classism interact perniciously, creating a housing market that disproportionately places Black women and their children at the highest risk for eviction.^10,11,77^ The ramifications of historical redlining and present-day systems and policies that perpetuate racial residential segregation, housing discrimination, biased tenant screening,^78,79^ and exploitative rental pricing^80^ craft and consolidate communities with concentrated housing insecurity. That Black mothers and their children bear a disproportionate eviction burden necessitates an explicitly intersectional, community-based approach to eviction protection, antipoverty advocacy, and reproductive justice.^78,79^

### Limitations

The results from this systematic review should be interpreted in the context of several limitations. First, the identified studies largely considered nonoverlapping outcomes, thus limiting our ability to observe replication across specific outcomes or conduct meta-analyses. Second, null findings are likely underrepresented due to publication bias. Third, eviction is a prevalent but evidently understudied stressor, resulting in a small number of articles. Fourth, the studies in our review measured eviction exposure at multiple scales ranging from direct individual experiences to county-level eviction rates. This was advantageous because individual- and neighborhood-level evictions likely affect health through distinct mechanisms worthy of independent investigation. However, quantifying the quality of evidence from various scales of analysis was difficult, given the inherent limitations of aggregated exposure and covariate data.

## Conclusions

The studies identified in this systematic review suggest that eviction exposure was associated with various adverse pediatric health outcomes, including preterm birth, low birthweight, lower neuropsychological scores, and worse parent-rated child health. An estimated 1 in 7 children nationally, and 1 in 4 children residing in poor households, will experience an eviction before 15 years of age.^81^ Preventing evictions is an urgent step toward health equity. In the context of recent, unprecedented yet temporary housing policy changes, these findings suggest that it is time to enact policies that prevent rental evictions, particularly among economically and racially marginalized communities who are disproportionately affected by the rental housing crisis.^58^

## Notes

Supplement 1. **eAppendix 1.** Search Strategy **eAppendix 2.** Newcastle-Ottawa Quality Assessment Scale for Cohort Studies

Supplement 2.
Data Sharing Statement
