# Supplementary material for: Evictions and Infant and Child Health Outcomes: A Systematic Review
Source: JAMA Netw Open. 2023 Apr 11;6(4):e237612. doi: 10.1001/jamanetworkopen.2023.7612 (PMC10091158; doi:10.1001/jamanetworkopen.2023.7612)
Supplement: Supplement 1. — eAppendix 1. Search Strategy eAppendix 2. Newcastle-Ottawa Quality Assessment Scale for Cohort Studies [file jamanetwopen-e237612-s001.pdf]

Supplemental Online Content

Ramphal B, Keen R, Okuzuno SS, Ojogho D, Slopen N. Evictions and infant and child health outcomes: a systematic review. *JAMA Netw Open*. 2023;6(4):e237612. doi:10.1001/jamanetworkopen.2023.7612

eAppendix 1. Search Strategy
eAppendix 2. Newcastle-Ottawa Quality Assessment Scale for Cohort Studies

This supplemental material has been provided by the authors to give readers additional information about their work.

© 2023 Ramphal B et al. *JAMA Network Open.*

eAppendix 1. Search Strategy

PubMed – 09/25/2022

(eviction[TIAB] OR "housing loss"[TIAB]) AND ("Infant"[Mesh] OR "Child"[Mesh] OR "Adolescent"[Mesh] OR "child"[tiab] OR "adolescent"[tiab] OR "youth"[tiab]) AND (English[lang])

Results: 79 articles

Web of Science databases (Science Citation Index Expanded (ISI), Social Sciences Citation Index (SSCI) and SciELO)

((TS=(eviction) OR TS=("housing loss")) AND (TS=(child) OR TS=(children) OR TS=(youth) OR TS=(adolescent) OR TS=(adolescence) OR TS=(infants)))

Results: 207 articles

APA PsycINFO

(eviction OR "housing loss") AND (infant OR child* OR adolesc* OR youth)

Results: 77 articles

© 2023 Ramphal B et al. *JAMA Network Open.*

eAppendix 2. Newcastle-Ottawa Quality Assessment Scale for Cohort Studies

Manual for its use in *Housing Eviction & Child Health* project
Adapted from http://www.ohri.ca/programs/clinical_epidemiology/oxford.asp.

Instructions:

- There are 8 items listed below that guides the process in assessing the quality of each article
- A study can be awarded a maximum of one star (or asterisk) for each numbered item
- When the study meets the threshold indicating a "good" design/ method (this is reflected by an asterisk below), give that study one star for that criterion in the excel spreadsheet. For criterion 5, "Comparability", the study could receive up to two stars.

Selection

1) Representativeness of the exposed cohort

a) truly representative of the average child experiencing eviction in the geographic location * (e.g., population-based studies)
b) somewhat representative of the average child experiencing eviction in the geographic location
c) selected group of users (e.g., hospital sample, children of parents with a chronic health condition)
d) no description of the derivation of the cohort

2) Selection of the non exposed cohort

a) drawn from the same geographic location as the exposed cohort *
b) drawn from a different source/geographic location but matched on age and sex *
c) drawn from a different source/geographic location
d) no description of the derivation of the non exposed cohort

3) Demonstration that outcome of interest was not present at start of study

a) Longitudinal and screened out OR controlled for health outcome at baseline*
b) Birth outcome *
c) Cross-sectional (not a birth outcome)

Comparability and Ability to make inferences at the individual

4) The subjects in different outcome groups are comparable, based on the study design or analysis. Confounding factors are controlled. (*up to 2 stars can be awarded*)

a) The study controls for basic characteristics, including household SES*
b) The study uses robust methods to address potential confounding, including propensity scores, fixed effects, marginal structural models, etc.*
c) No controls

Outcome

5) Assessment of outcome

a) Non self-report or parent-report (E.g., "measured" by research team)*
b) Record linkage to individual-level data*
c) Parent- or self-report using a validate instrument*
d) No description OR single item self or parent reported health

© 2023 Ramphal B et al. *JAMA Network Open.*

e) aggregated outcomes assessed at the area-level

6) Statistical test

a) The statistical test used to analyze the data is clearly described and appropriate, and the measurement of the association is presented, including confidence intervals OR the probability level (p value). *

b) The statistical test is not appropriate, not described, or incomplete.

7) Adequacy of follow up of cohorts or completeness of data

a) complete follow up OR no missing data - all subjects accounted for*

b) subjects lost to follow up unlikely to introduce bias results, or careful methods to account for bias that could be caused by attrition or missing data (e.g., multiple imputation or inverse probability weighting to account for attrition) *

c) no analyses to account for those lost or no statement

+Adapted from: Wells, G. A, Shea, B., O'Connel, D. et al. The Newcastle-Ottawa scale (NOS) for assessing the quailty of nonrandomised studies in meta-analyses. http://www ohri ca/programs/clinical_epidemiology/oxford htm 2009 Feb 1
Note – some adaptations based on cross-sectional version of Newcastle-Ottawa rating scale

© 2023 Ramphal B et al. *JAMA Network Open.*
