# Supplementary material for: Evictions and Infant and Child Health Outcomes: A Systematic Review
Source: JAMA Netw Open. 2023 Apr 11;6(4):e237612. doi: 10.1001/jamanetworkopen.2023.7612 (PMC10091158; doi:10.1001/jamanetworkopen.2023.7612)
Supplement: Supplement 2. — Data Sharing Statement [file jamanetwopen-e237612-s002.pdf]

Data Sharing Statement

Ramphal. Evictions and Infant and Child Health Outcomes. *JAMA Netw Open*. Published April 11, 2023. doi:10.1001/jamanetworkopen.2023.7612

Data
Data available: No

Additional Information
Explanation for why data not available: There is no data for this study.
